# Digital Intervention for Problematic Smartphone Use

**DOI:** 10.3390/ijerph182413165

**Published:** 2021-12-14

**Authors:** Sarah Kent, Ciara Masterson, Raian Ali, Christine E. Parsons, Bridgette M. Bewick

**Affiliations:** 1 School of Medicine, University of Leeds, Leeds LS2 9JT, UK; c.masterson@leeds.ac.uk; 2 College of Science and Engineering, Hamad Bin Khalifa University, Doha, Qatar; raali2@hbku.edu.qa; 3 Interacting Minds Center, Department of Clinical Medicine, Aarhus University, 8000 Aarhus, Denmark; christine.parsons@cas.au.dk

**Keywords:** students, smartphone use, e-health, digital intervention, case series, smartphone addiction, digital addiction

## Abstract

Smartphones have become the primary devices for accessing the online world. The potential for smartphone use to become problematic has come into increasing focus. Students and young adults have been shown to use their smartphones at high rates and may be at risk for problematic use. There is limited research evaluating interventions for problematic smartphone use. The present research aimed to develop and evaluate a digital intervention for problematic smartphone use in a student population. A mixed-method case series design was used. The participants were 10 students with mild–moderate dependency on the online world (measured via a self-report questionnaire). An intervention comprising goal setting, personalised feedback, mindfulness, and behavioural suggestions was delivered via a smartphone application. Time spent on smartphones was measured objectively through the same application. Changes in problematic technology use, wellbeing, mindfulness, and sleep were also evaluated. The findings indicate that the intervention resulted in a reduction in self-reported problematic smartphone use, but not screen time. The findings also indicate that over the course of participation, there was a positive influence on wellbeing, online dependency, mindfulness, and sleep. However, the mechanisms of change could not be determined. The study provides preliminary evidence that a light-touch, smartphone-delivered package is an acceptable and effective intervention for students wishing to better manage their problematic smartphone use.

## 1. Introduction

Smartphones are now the primary devices for accessing the online world [1]. Smartphones are a developing technology, and much of our understanding of how people interact with the online world pre-dates their common use, but smartphones have unique qualities when compared to other digital devices with internet connectivity. Smartphones are ubiquitous devices that have the potential to always be on and constantly connected; they are compact and afford flexibility and ease of use. The persuasive qualities of smartphones increase the frequency of use and encourage checking habits [2].

Smartphone use is considered to become problematic when it begins to interfere with health, wellbeing, and the ability of an individual to function in daily life [3]. Although technological addictions are not considered to be a diagnosis within diagnostic manuals (see DSM-V [4]), they are characterised by salience, compulsive use, withdrawal symptoms, escapism, negative outcomes, mood regulation, and social comfort [5]. Problematic smartphone use is closely linked to ‘generalised internet addiction’—which is understood to differ from problems with specific elements of online usage—characterised by non-directed time wasting, often with overuse of multiple online applications [6].

Almost all young adults use a smartphone, and it is their device of choice for electronic/online activities [1]. Young adults use digital devices more regularly and spend more time on them compared to other age groups [7]. Given this context, it is pertinent to pay attention to the role that smartphones will play in university students’ lives and to the potential for resultant problems. Students have higher prevalence rates of internet addiction [8], and it is likely that online use is particularly encouraged within this population (e.g., by peers socially, by universities/colleges for academic purposes). Students are now reliant on the internet and, by extension, their smartphones as the quickest and most efficient way of accessing information. The embedded nature of internet use is not necessarily problematic, but it does increase one’s reliance upon it and may increase vulnerability to problematic use. In addition, the perceived normalisation of heavy internet use reduces the likelihood of individuals recognising when their smartphone use becomes problematic.

There is preliminary evidence that students are ready and willing to make changes in their digital use [9] and that they are interested in being supported in regulating their usage [10]. There is cautious support for the role of e-health interventions in supporting the development of healthy interactions with the digital world and reducing problematic use [11]. The aim of such interventions is to control or regulate use, rather than to promote abstinence; it is therefore appropriate to use smartphones themselves as a vehicle for intervening in problematic smartphone use.

There are promising indications that behavioural interventions can be effective in modifying problematic digital use (e.g., cognitive behavioural therapy [12,13]). While there is a relative paucity of evidence evaluating the active ingredients of e-health interventions for problematic digital use, existing evidence suggests that goal setting [14], personalised feedback [10,15], and mindfulness [16] are likely to contribute to significant behavioural change.

The current research evaluated the acceptability and effectiveness of a light-touch, smartphone-delivered e-health intervention designed to reduce problematic smartphone use in undergraduate students in the UK.

The intervention was designed with goal setting, personalised feedback, mindfulness, and behavioural phases. In order to measure its effectiveness, the study explored changes in smartphone and online use, wellbeing, and related constructs across the phases. It was hypothesised that improvements would be observed in screen time, problematic technology use, mindfulness, sleep, and wellbeing if the intervention was successful.

## 2. Materials and Methods

### 2.1. Design

A single-case experimental design (SCED) was used to investigate the impact of the intervention with both quantitative and qualitative data collected from each participant. This method is particularly well suited to testing new e-health interventions [17]. In an SCED, each participant provides data for the control condition (i.e., baseline) and intervention. Systematic manipulation of the independent variable (i.e., the intervention) was achieved by randomly allocating the order of treatment phases. The multiple data collection points in each phase strengthened the design, and the data collected included: real-time objective measurement, regular standardised self-report measures, and an interview.

### 2.2. Participants

A convenience sample of ten undergraduate students was recruited, which was a sample of sufficient size for tentative conclusions regarding the efficacy of the intervention [18,19]. Eligibility for participation in the case series was assessed via an online survey. Participants were required to be undergraduate students who presented with problematic online use (as measured by the Internet Addiction Scale [20,21]), to be contemplating change (indicated by survey questions based on the stages of change model [22]), and to be Android smartphone users (owing to the application being used). Participants were aged between 18 and 31, with a gender ratio of 9:1 (F:M). All participants were white European and varied in relation to faculty, school, and year of study (see Table 1). Participants were assigned a pseudonym (a colour), which was used throughout analysis and reporting. The case series participants were reimbursed up to a total of 30 GBP for their participation in the study (dependent upon duration of participation).

### 2.3. Measures

**Figure 1:**
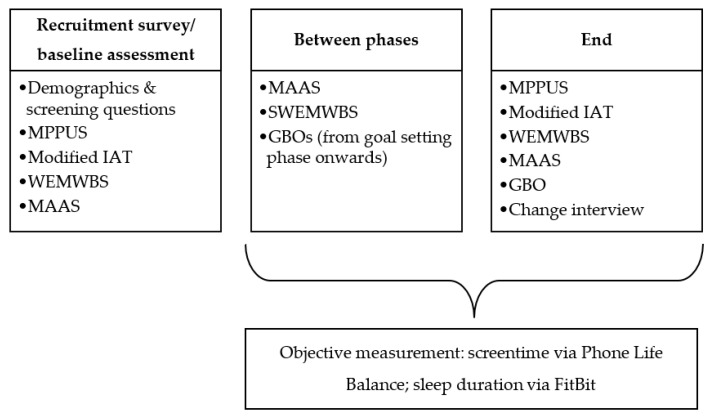
An outline of the stages at which each of the measures was completed.

Problematic technology use, wellbeing, sleep, and mindfulness were measured across the intervention via a combination of objective and self-reported measurements. Each method and tool of measurement is detailed below, and the points of measurement are detailed in Figure 1.

#### 2.3.1. Technology Use

##### Phone Life Balance 

Phone Life Balance [23] is an Android smartphone application; an adapted version of this application was used in the present research, which collected phone use data and allowed for messages to be sent to the user. It collected data on which apps were being used and for how long.

##### Mobile Phone Problem Use Scale (MPPUS) 

The MPPUS [24] is a 27-item measure that uses a 10-point Likert scale, ranging from 1 (“not true at all”) to 10 (“extremely true”), to measure the extent to which an individual’s phone use is problematic. Total scores range from 27 to 270, with higher scores indicating greater levels of problematic use. There are no cut-off scores published for this measure. Good internal consistency has been found [24].

##### Modified Internet Addiction Test (IAT) 

The IAT [20,21] is a 20-item measure that uses a 6-point Likert scale, ranging from 0 (“does not apply”) to 5 (“always”), to measure the severity of an individual’s dependency on the online world. The following categories of online dependency were identified by the developer: 0–30 = Normal; 31–49 = Mild; 50–79 = Moderate; 80–100 = Severe [8].

The IAT shows good reliability within student populations [25]. Slight language modifications were made to some items to reflect the current ways in which the online world is used; the modifications did not change the meaning of the questions.

#### 2.3.2. Wellbeing and Related Constructs

##### The Warwick Edinburgh Mental Wellbeing Scale (WEMWBS) 

The WEMWBS [26] is a 14-item measure scored on a 5-point Likert scale, ranging from 1 (“none of the time”) to 5 (“all of the time”), to measure an individual’s mental wellbeing. Total scores range from 14 to 70, with higher scores indicating greater levels of mental wellbeing. There are no cut-off scores for this measure, as wellbeing is not a clinical construct. The measure has been shown to have good psychometric properties [26].

##### The Short Warwick Edinburgh Mental Wellbeing Scale (SWEMWBS) 

The SWEMWBS [27] is a measure made up of 7 items taken from the WEMWBS, which is scored on the same 5-point Likert scale, to measure an individual’s mental wellbeing. Total scores range from 7 to 35, and as above, higher scores indicate greater levels of wellbeing.

##### The Mindful Attention Awareness Scale (MAAS) 

The MAAS [28] is a 15-item measure scored on a 6-point Likert scale, ranging from 1 (“almost always”) to 6 (“almost never”), to measure an individual’s level of mindfulness. To score the MAAS, the mean of all items is calculated, meaning that final scores range from 1 to 6, with higher scores indicating greater levels of mindfulness. There are no cut-off scores for this measure. The validity of the measure has been demonstrated [29].

##### FitBit Charge 2

The FitBit Charge 2 is a wrist-worn activity watch that tracks activity, heartrate, and sleep. Only the sleep data were used for analysis in the present research.

#### 2.3.3. Idiosyncratic Measurement

##### Goal-Based Outcomes (GBOs)

GBOs [30] measure an individual’s progress towards any goal(s) they have identified by using a 0–10 rating scale (10 indicating that the goal has been fully achieved).

##### Client Change Interview

A client change interview [31] is a semi-structured interview designed to evaluate the therapy process and identify changes that have occurred; this was adapted for the present research. The interview was designed to explore any changes that clients/participants perceived to have occurred and to what they attribute these changes.

### 2.4. Procedure

#### 2.4.1. Baseline

At the start of the study, all self-reported measures were completed. Following this, over a two-week period, baseline sleep and smartphone use data were collected via the respective device/application used to obtain objective data (data collection continued throughout the intervention).

#### 2.4.2. Intervention

For the purpose of this study, the intervention was delivered manually. A template for content and timing of messages was utilised to ensure that the intervention was standardised and could be automated in the future if found to be effective. This template enabled messages to be personalised while still providing an overarching structure for the intervention, and it supported messages being delivered at pre-determined intervals.

To begin the intervention, participants were invited to set goals. Goal options were provided, one relating to their smartphone use (e.g., “be less distracted by my smartphone”) and another relating to improvement of other behaviours (e.g., studying). Participants could also select “other” and use a free text box. Achievement of goals was measured using goal-based outcomes.

The intervention consisted of three further phases (see Table 2 for more details). Participants received a message each day for seven consecutive days within each of these phases; between these phases, the outcome measures were completed. All participants received a phase of personalised feedback following the goal setting; the next two phases were mindfulness and behavioural suggestions (the order of which was randomised). Following the intervention phases, there was a two-week consolidation phase during which participants were asked to continue with suggestions that they had found useful. All messages were delivered to participants’ smartphones via either the Phone Life Balance app or WhatsApp (an online messaging platform that was utilised where there was an incompatibility issue with Phone Life Balance).

### 2.5. Final Assessment and Change Interview

The participants completed all self-reported measures after the consolidation phase. Following this, change interviews were completed based on Elliot’s [31] protocol.

### 2.6. Analysis

To address the hypotheses, a visual analysis and reliable change analysis were used alongside the qualitative data obtained during the change interviews. The visual analysis explored changes between phases and over the course of the study [32]. Medians and interquartile ranges were calculated for screen time data for each phase. To measure overall change in the objective data the medians calculated for the daily screen time and sleep duration during the consolidation phase were compared to those calculated for the baseline phase.

The difference between the pre- and post-score for each of the standardised self-reported measures for each participant was calculated to obtain a change score. The reliable change criteria for each standardised measure were calculated using an online calculator. If the change score exceeded the reliable change criteria, this was considered to be a reliable change.

The data obtained via the change interview and the goal-based outcomes were utilised to support the understanding and interpretation of the other data.

## 3. Results

### 3.1. Overall Engagement and Change

No participants chose to withdraw from the study, and all said that they would recommend the intervention to others. All participants engaged with the intervention, although none reported engaging with every message received. Of the 21 messages sent to each participant, the participants reported engaging with between 9 and 18 (median = 13).

**Figure 2:**
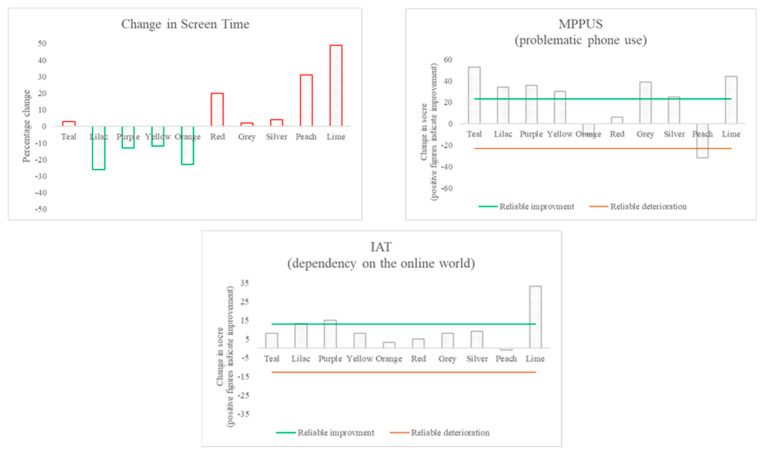
Changes in technology use: the difference between pre- and post-measurement for each participant.

**Figure 3:**
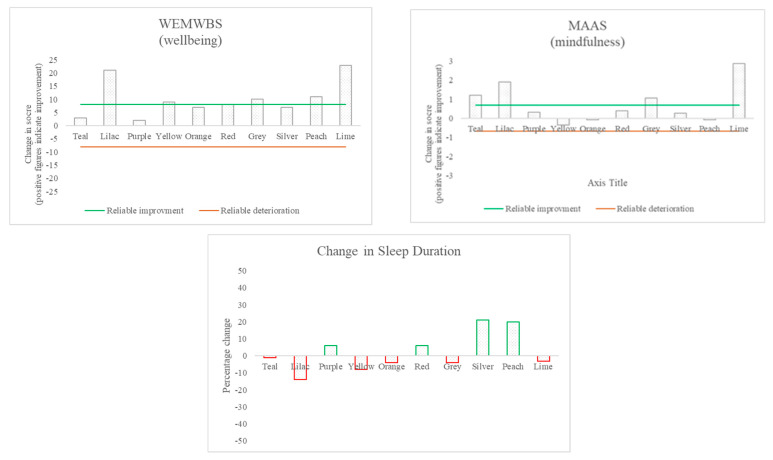
Changes in wellbeing and related constructs: the difference between pre- and post-measurement for each participant.

**Figure 4:**
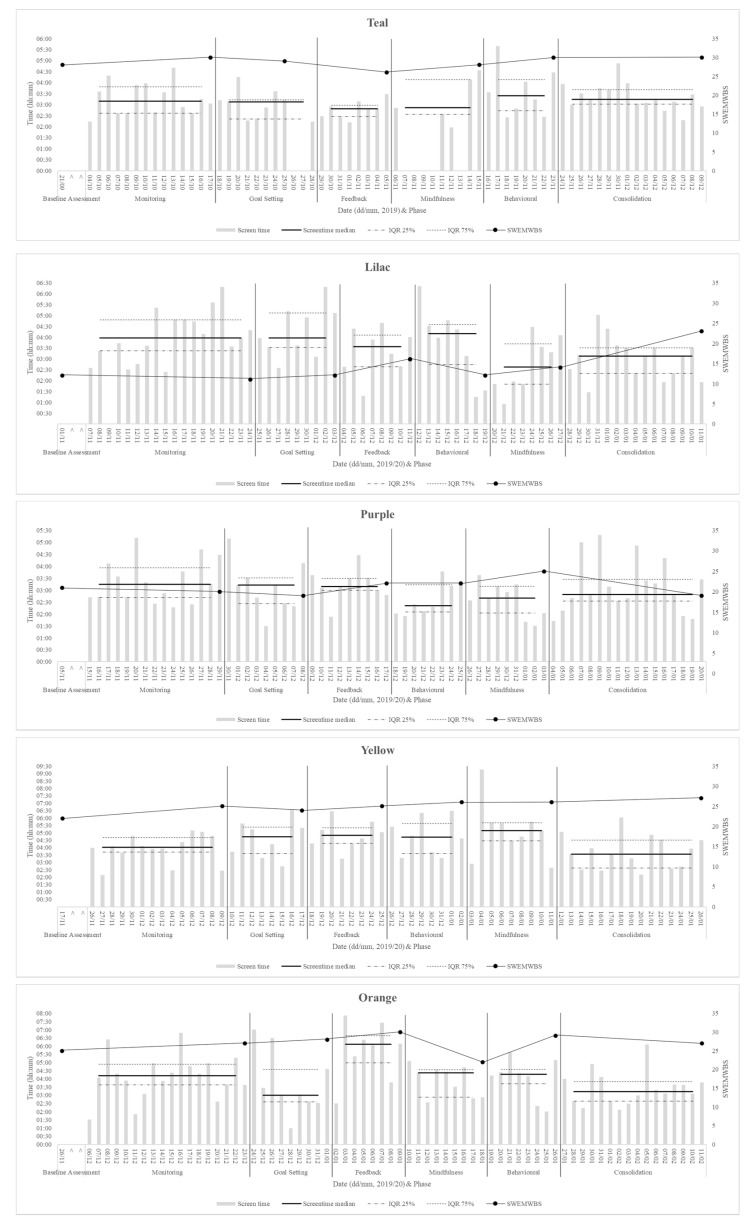

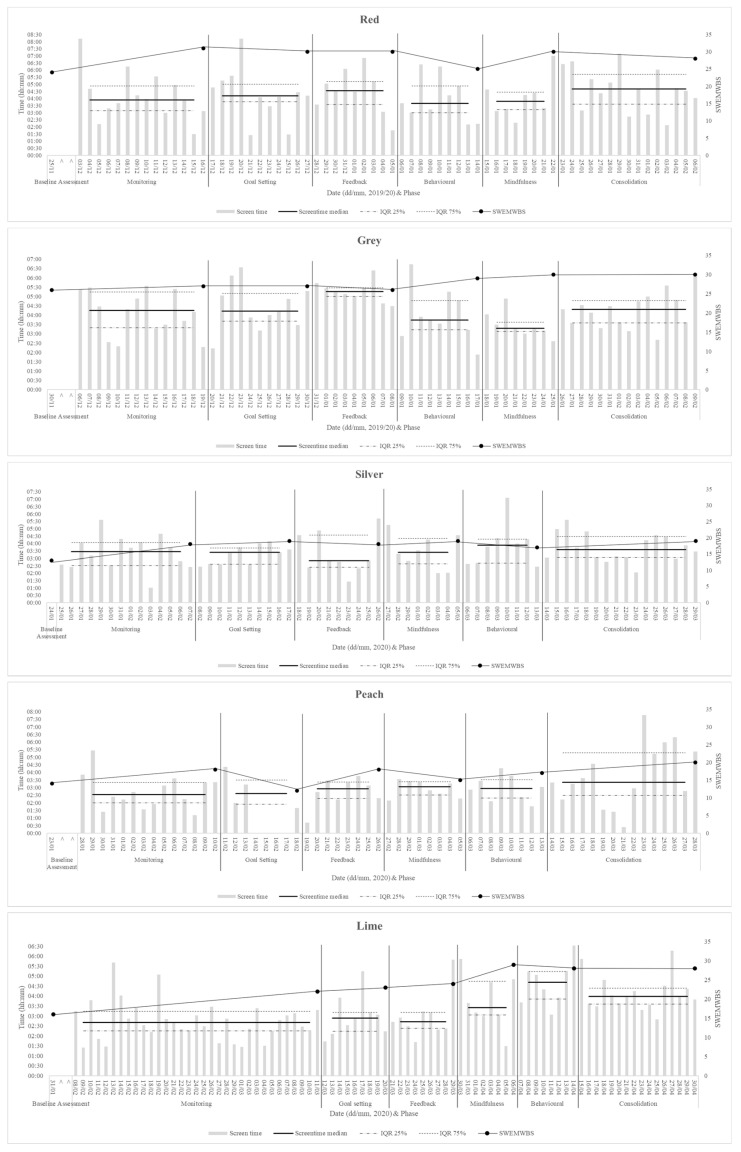
Total daily screen time throughout the participation in the study for each participant, along with the screen time median and interquartile range and the SWEMWBS scores for each phase.

Most participants evidenced improvement in problematic smartphone use, as described below, and all showed an improvement in wellbeing, although a clear association between the two could not be determined (see Table 3, Figure 2, Figure 3 and Figure 4).

Although overall change was evidenced, the data do not provide insight into the impact of each individual intervention phase. The data collected throughout the study show fluctuations in both screen time and wellbeing, with no consistent patterns across participants (see Figure 4).

### 3.2. Changes in Technology Use

The pre- and post-outcome measure scores were used to determine the overall change in problematic smartphone use (see Table 3 and Figure 2). Eight of the ten participants showed improvements in problematic phone use (according to the MPPUS), with all but one of those improvements meeting the reliability criteria. One participant showed a reliable deterioration on the MPPUS; in the interview, this participant reported finding the behavioural suggestions too challenging. The IAT showed improvements in dependency on the online world for nine of the ten participants, with three of those changes meeting the criteria for reliability. When each participant’s median screen time per day during the consolidation phase was compared to the median during the baseline phase, a reduction in screen time was seen for four participants, whilst three showed very small percentage increases and three showed larger percentage increases (see Figure 2 and Figure 4). These changes in screen time did not appear to be associated with changes in problematic use, and participants often reported changes to be circumstantial (e.g., related to the COVID-19 lockdown, during exam periods, or over the university holidays). However, there were three participants who made improvements on all measures associated with smartphone use.

The results here suggest that the MPPUS and the IAT measure different concepts: although the IAT showed improvements over the course of the intervention, these were less pronounced than those made on the MPPUS for most participants. Further, the results also suggest that perceived problematic phone use does not equate to screen time, indicating that problematic use more likely relates to the way the phone is used and/or the relationship with it than the time spent using it.

The collective results demonstrate that for almost all participants, there was an improvement in problematic phone use and dependency on the online world (as indicated by the standardised measures).

### 3.3. Changes in Wellbeing and Related Constructs

Improvements in wellbeing (as measured by the WEMWBS) can be seen for all ten participants; six of these reached the level for reliability (see Figure 3). Three participants spoke of wellbeing-related changes in the interviews as key changes that had occurred. The changes in wellbeing did not occur at a consistent time point in the intervention across participants (see Figure 4).

There were improvements in mindfulness (as measured by the MAAS) for seven of the ten participants; four of those changes reached the criteria for reliability. Increased awareness and/or mindfulness were also spoken about by many of the participants in the change interviews.

The sleep data recorded via the FitBit indicated that for most participants, there were big fluctuations in sleep duration (often ranging from as little as 2–4 h to as much as 10–12 h) and no overall significant changes throughout the course of the study. Eight of the ten participants self-reported an improvement in their sleep, however. Two of those participants identified improvements in sleep as a key change during the change interview.

The results indicate that, overall, there was a positive impact upon wellbeing and levels of mindfulness over the course of the study (although these changes were often small). The survey data are suggestive that some improvements were made in participants’ sleep, but the extent of these changes is unclear.

### 3.4. The Relationship between Changes in Technology Use and Other Changes

There was no clear relationship evidenced between the changes in the different measures when analysing the pre- and post-intervention data (see Figure 2 and Figure 3). Further consideration was paid to the relationship between wellbeing and smartphone use (see Figure 4). Across participants, there was no consistent pattern in screen time change over the course of the intervention or in how it related to changes in wellbeing scores. Additionally, two participants showed improvements in wellbeing, but a deterioration in problematic smartphone use.

Overall, the association between the different outcomes cannot be determined in the current study. It was expected that screen time would be an accurate measure of problematic use; however, given that the results suggest otherwise, the available data do not allow for the relationship between problematic smartphone use and wellbeing to be understood.

### 3.5. Participants’ Perspectives on Change

Over the course of the study, all participants reported an improvement in the achievement of their self-identified goals (see Table 4). These improvements were seen in goals relating to both changing their smartphone use and improving another area of their life impacted by their smartphone use (e.g., studying).

In the change interviews, participants were asked to identify any changes that had occurred over the course of the study. Participants reported between one and five changes, and all participants rated at least one of those changes as somewhat or very unlikely without the intervention. There was only one negative change reported, which was increased guilt relating to awareness of phone use. Five participants specifically reported a change related to increased awareness of phone use (e.g., Teal: “just giving me that awareness…to be like actually why, why are you going on it now…what, what purpose is it serving you?”). Although increased awareness was implicit in most of the change interviews, some participants described it as a contributor to other changes, rather than describing it as a change itself. Six participants described changes that related to phone use; these included reducing phone usage, having time away from their phone, and being less distracted by their phone. There was an emphasis on changing the way phones are used; for example: “it is understandable that people use it a lot…I think it’s more like high usage…that should be kind of focused” (Purple); “I’m getting better at using it for just the useful thing without getting distracted by other things” (Yellow); “improving my relationship with it is crucial” (Grey). Changes suggestive of increased mindfulness were listed by four participants and changes suggestive of improved wellbeing by three.

Participants gave specific examples of messages or ideas they had found useful, e.g., “definitely the ones to leave the phone in another room…or to try eating or to try do other activity without looking at your phone” (Orange). All participants expressed their intention to continue utilising some of the learning they had gained from the intervention.

These data support the changes observed in other measures and indicate that participants made changes that were meaningful to them. The data also provide evidence that participants attributed some of the changes they made to the intervention they received.

## 4. Discussion

### 4.1. Discussion of Findings

This case series evaluated an e-health intervention for problematic smartphone use for university students with mild to moderate levels of dependency on the online world. Based on the engagement of participants and responses to the change interview (all participants said they would recommend the intervention to others), there is evidence that the intervention is acceptable. There were significant improvements in measures of problematic phone use and wellbeing for the majority of participants; improvements were also seen in dependence on the online world and mindfulness for most participants.

Whilst there is evidence of improved wellbeing, mindfulness, and sleep over the course of the study, the relationship between these changes and changes in phone use could not be determined from the data collected. Some participants described a link between increased mindfulness and reduced problematic phone use, although the strength and nature of this relationship cannot be understood with the available data. This suggests that further research into the mechanisms of change is required.

The intervention package delivered in the present study was derived from the research focusing on problematic use of technology and the internet, as well as drawing upon what we know about interventions with other problematic behaviours. The package comprised multiple phases (goal setting, personalised feedback, mindfulness, and behavioural suggestions). The findings suggest that the intervention package was effective. The changes observed could not be attributed to any one phase; participants’ reflections suggested that the combined intervention was important.

Although the results showed evidence of reduced problematic or dependent technology use, they did not show a consistent change in screen time over the course of the study for the participants. Whilst this suggests that the intervention is effective in reducing problematic use, it also confirms that problematic use is a complex concept. For participants in this study, problematic use did not equate to time spent on their smartphones. In interviews, they highlighted their ability to control their phone use and the impact it had on other areas of their lives as more important than the time spent on their phones. As such, the MPPUS was considered to be the best measure of problematic use. Previous research regarding this relationship in students has resulted in mixed findings; some studies found that the severity of problematic smartphone use was related to screen time, but not frequency of use [33,34], whilst other studies found a correlation only with frequency of use [35,36]. This confirms that problematic phone use is multifaceted and is most likely related to screen time, frequency of use, perceived control, and impact.

People are often unaware of when their technology and/or online use becomes problematic [37,38], suggesting that increasing awareness might be important. Until very recently, there had been no evaluation of the impact of offering feedback on problematic smartphone use. A recent study found that offering feedback alone had no impact upon the level of problematic use and resulted in only a small (not statistically significant) reduction in screen time [33]. Although these preliminary findings suggest that feedback is ineffective, it may be the case that increased awareness facilitates progress through the stages of change [22], thus increasing the likelihood of future behavioural change. In the present study, all participants reported an awareness that their phone use was problematic prior to participation; this awareness was a prerequisite, given that somebody must recognise a problem in order to want to make a change. Despite all participants in the current study having this prior awareness, many reported increased awareness over the course of the intervention, with some specifically linking this to the personalised feedback messages they received. This suggests that whilst participants had an awareness, they were perhaps under-aware [39], especially given that people tend to underestimate their smartphone usage [36]. The current findings suggest that increased awareness often enabled or facilitated behavioural change.

The mindfulness suggestions were aimed at bringing focus into the present moment, whilst the behavioural suggestions aimed to bring it away from the phone. Although we are unable to identify how the separate intervention phases impacted behaviour differently, we do know that both online and offline behaviours were influenced for most participants. The importance of focusing on online and offline behaviours has been emphasised in the internet addiction literature [40], and it also fits with the concept of ‘approach goals’ (moving towards something positive) being influential [41]. Making changes in both areas was important for the participants in the present study.

A question remains about the nature of problematic use and the likely characterisations of different presentations, including whether it is a primary problem or secondary to something else (e.g., anxiety or depression). Some authors have questioned this and suggested that formulation of an individual’s presenting problem is important [3], and others have highlighted the importance of identifying and managing triggers for use [42]. Whilst these are likely to be beneficial, in the present study, they did not appear to be necessary for significant changes to be made. Some participants did reflect on the need to access additional support for other psychological difficulties, but this did not prevent them from engaging with and benefiting from the problematic smartphone use intervention.

### 4.2. Strengths and Limitations

It is a strength that the case series design of this study enabled a large amount of data (using multiple and frequent measurement) to be collected for each participant, facilitating a thorough analysis of change [43]. However, the results revealed that screen time was not the best measure of problematic phone use, thus limiting the conclusions that could be drawn and leading to greater reliance on the change interview data. The mixed-method design added weight to the validity of the conclusions through the gathering of qualitative data from participants during the change interviews to corroborate any conclusions drawn based upon the quantitative data. Despite evidence of change overall, it is unclear exactly when the changes occurred for the participants in the present study. The study was designed with a two-week monitoring phase, with the intention of participants becoming habituated to the monitoring application; it is possible that monitoring alone influenced participants’ relationships with their phones. Research aimed at reducing alcohol consumption in students has suggested that monitoring positively influences behavioural change, although there was additional benefit seen where students were also offered an intervention [44]. Further research is needed to explore whether this is true for problematic technology use.

No participants chose to withdraw from the study, which is a strength. Attrition can be considered to bias results [45], and so the low attrition rate reduced the chance of bias. It is also a strength that during the change interviews, participants were given the opportunity to reflect on events outside of the intervention that may have influenced changes [43], and that participants did attribute changes to the intervention itself.

As described in the method, for some participants, the intervention messages were delivered via WhatsApp. WhatsApp is a social media application; receiving the intervention messages via this platform may have increased the likelihood of users engaging with WhatsApp for other reasons.

The study was limited by only being available to Android users; this has the potential to impact the generalisability of the findings. The recruited sample lacked diversity overall, with nine of the ten participants being female and all being white and of European origin, meaning that the generalisability of the results is uncertain. Whilst single-case experiments have been accepted as demonstrating efficacy for an intervention, Chambless and Hollon [18] state the requirement for evidence from at least two research groups/sites in order to achieve the designation of ‘efficacious’; therefore, research with similar interventions in other student populations is needed.

## 5. Conclusions

The present study demonstrates that a new smartphone-delivered package with monitoring and intervention phases was effective for improving problematic smartphone use in students. However, the pattern of change in the current participants did not allow us to reach firm conclusions regarding the mechanism of change. There is some evidence that the intervention also had a positive impact upon dependency on the online world, mindfulness, wellbeing, and sleep. The relationship between these changes and those made in problematic phone use, as well as the relationship between problematic phone use and screen time, remains unclear.

The findings suggest that problematic smartphone use is best measured via a self-report tool (such as the MPPUS), which, in the present study, was only administered pre- and post-intervention. Future research administering the MPPUS at more regular time points may facilitate a better understanding of the change process and the relationships among problematic technology use, screen time, wellbeing, mindfulness, and sleep. Despite this, it appears that the intervention is both acceptable and effective, and it should therefore be offered to students who wish to make changes in their problematic smartphone use.

## Figures and Tables

**Table 1:** Participant demographics.

Demographic	Value	N (*n* = 10)	(%)
Gender	Female	9	(90)
	Male	1	(10)
Faculty of Study	Arts, Humanities, and Cultures	3	(30)
	Engineering and Physical Sciences	4	(40)
	Medicine and Health	2	(20)
	Social Sciences	1	(10)
Age	18–21	7	(70)
	22–25	2	(20)
	26–29	-	-
	30–33	1	(10)
Undergraduate Year of Study	1	1	(10)
	2	2	(20)
	3	4	(40)
	4	1	(10)
	5	1	(10)
	Other	1	(10)

**Table 2:** Example intervention messages.

Phase	Example Messages
Personalised feedback	“You spend 3 h a day on your phone, over a year this would be over 45 whole days.” Image: a bar chart showing average daily screen time for weeks 1–4
Behavioural suggestions	“You said that leisure time is important to you. Plan an activity, whilst doing this set your phone to ‘do not disturb’ and place it out of sight.”
Mindfulness suggestions	“Pick an everyday activity and focus all of your attention on this activity while you do it. Your mind might wander but bring it back to the activity. You can use your senses like the activity yesterday.”

**Table 3:** Pre- and post-intervention scores on MPPUS, IAT, MAAS, and WEMWBS.

	MPPUS		IAT		MAAS		WEMWBS	
Participant	Pre	Post	Pre	Post	Pre	Post	Pre	Post
Teal	134	81 ^a^	40	32	4.2	5.4 ^a^	54	57
Lilac	138	104 ^a^	61	48	2.6	4.5 ^a^	22	43 ^a^
Purple	178	142 ^a^	59	44 ^a^	1.67	2	38	40
Yellow	171	141 ^a^	47	39	4.13	3.79	43	52
Orange	130	140	33	30	3.93	3.87	49	56
Red	149	143	57	52	3.93	4.33	48	56 ^a^
Grey	160	121 ^a^	50	42	4.47	5.53 ^a^	48	58 ^a^
Silver	144	119 ^a^	58	49	2.67	2.93	29	36
Peach	91	123 ^b^	35	36	3.07	3 ^b^	29	40 ^a^
Lime	142	98 ^a^	41	8 ^a^	1.53	4.4 ^a^	33	56 ^a^

^a^ Reliable improvement; ^b^ Reliable deterioration.

**Table 4:** Changes in goal-based outcomes.

Participant	Goal 1 (Change in Smartphone Use)	Change in Goal 1 Achievement Rating	Goal 2 (Area of Life Wanting to Improve)	Change in Goal 2 Achievement Rating
Teal	Be less distracted by	+8	Leisure time	+4
Lilac	Reduce time spent overall	+8	Relationships with family	+5
Purple	Check less often	+5	Studying	+1
Yellow	Be less distracted by	+4	Studying	+4
Orange	Reduce time spent overall	+2	Studying	+2
Red	Reduce time spent on social media	+7	Relationships with family	+4
Grey	Be less distracted by	+2	Studying	+2
Silver	Reduce time spent overall	+2	Studying	+1
Peach	Be less distracted by	+1	Studying	+1
Lime	Be less distracted by	+5	Leisure time	+6

## Data Availability

The data presented in this study is available in full here: Digital Intervention for Problematic Smartphone Use in Undergraduate University Students: A Systematic Case Series—White Rose eTheses Online.
